# Determinants of Brain Atrophy in People Living with HIV: The Role of Lifestyle, Demographics, and Comorbidities

**DOI:** 10.3390/jcm14134430

**Published:** 2025-06-22

**Authors:** Mihai Lazar, Cristina Emilia Chitu, Daniela Adriana Ion, Ecaterina Constanta Barbu

**Affiliations:** 1 Faculty of Medicine, University of Medicine and Pharmacy Carol Davila, No. 37, Dionisie Lupu Street, Sector 2, 020021 Bucharest, Romania; mihai.i.lazar@gmail.com (M.L.); daniela.ion@umfcd.ro (D.A.I.); ecaterina.barbu@umfcd.ro (E.C.B.); 2 National Institute for Infectious Diseases Prof. Dr. Matei Bals, No. 1, Calistrat Grozovici Street, Sector 2, 021105 Bucharest, Romania

**Keywords:** brain atrophy, structural MRI, brain morphometry, gray matter, white matter, PLWH, HAND, diabetes

## Abstract

**Background/Objectives:** This study aims to investigate the influence of demographic, behavioral, anthropometric, and comorbid factors on brain atrophy in people living with HIV (PLWH). **Methods:** We conducted a cross-sectional study involving 121 HIV-positive patients, stratified into two groups, those with and without brain atrophy (BA). For each participant, we recorded demographic data, smoking status, physical activity levels, disease and treatment duration, and comorbidities. BA was quantitatively assessed using MRI-derived volumetric measurements of 47 cerebral substructures. **Results:** Patients with BA exhibited significantly reduced gray matter (GM) and white matter (WM) volumes alongside increased cerebrospinal fluid volumes, both in absolute and percentage measurements. WM atrophy was most pronounced in the frontal, parietal, and temporal lobes, with relative sparing of the occipital lobe. GM atrophy predominantly affected the basal ganglia (notably, the thalamus and putamen) and cortical regions, including the hippocampus, frontal, and parietal lobes. Significant positive correlations were observed between BA and both smoking status (pack–years) and disease duration, while physical activity demonstrated an inverse relationship (higher atrophy risk in those with less than 30 min of daily continuous walking). Non-adherence to antiretroviral therapy (ART) was also associated with BA. Among comorbidities, type 2 diabetes and HIV-associated neurocognitive disorders (HAND) showed the strongest associations with BA. **Conclusions:** Brain atrophy in PWH is correlated with smoking, physical inactivity, and the duration of HIV infection. Comorbid conditions, such as type II diabetes and HAND, amplify the risk for BA. We consider that early lifestyle interventions and optimized ART may mitigate the neurodegeneration process.

## 1. Introduction

Despite remarkable advancements in combination antiretroviral therapy (cART), HIV infection remains a chronic condition associated with a wide range of complications, including neurocognitive decline and brain atrophy. As life expectancy for people living with HIV (PLWH) approaches that of the general population, age-related comorbidities have become increasingly prevalent and clinically significant [1]. Among these, brain atrophy—characterized by the loss of neuronal volume and integrity—has emerged as a key concern due to its association with HIV-associated neurocognitive disorders (HAND) and increased risk of dementia [2].

Globally, an estimated 39.9 million people were living with HIV in 2023, with 1.3 million people becoming newly infected with HIV, and more than 30.7 million accessing antiretroviral therapy [3]. As the demographics of PLWH shifts toward older age groups, understanding the interaction between HIV infection, aging, and other modifiable and non-modifiable risk factors becomes imperative. Neuroimaging studies have consistently demonstrated structural brain changes in PLWH, including cortical thinning, reduced gray and white matter volumes, and alterations in subcortical regions, even in individuals with virologically suppressed HIV [4,5]. An accelerated aging process was observed in PLWH, which consists of developing age-related diseases at younger ages compared to the general population [6].

Growing literature data suggests that lifestyle and demographic factors may influence the extent of brain atrophy in PLWH. For instance, while cigarette smoking has been independently associated with reduced brain volumes and worsened neurocognitive performance in HIV-positive cohorts [7], physical activity has demonstrated neuroprotective effects in PLWH [8]. Sex-related differences have been observed as well, with some studies indicating that women may experience different patterns or trajectories of neurocognitive impairment compared to men, potentially due to hormonal or immunological differences [9].

Anthropometric variables, such as weight, height, and body mass index (BMI), have been less extensively studied in the context of brain atrophy in HIV, yet emerging evidence suggests they may play a role through mechanisms such as inflammation, metabolic syndrome, or increased cerebrovascular risk [10]. High cumulative BMI has been found to be associated with smaller brain volume, larger white matter lesions, and abnormal microstructural integrity in the general population and may have similar implications in PLWH [11].

The duration of HIV infection and length of antiretroviral treatment also appear to be critical in modulating neuroanatomical changes. Longer disease duration has been correlated with more pronounced atrophy, possibly reflecting chronic immune activation, neuroinflammation, and legacy effects of earlier, less effective ART regimens [12]. Meanwhile, earlier and sustained cART has been associated with better preservation of brain structure, though some studies indicate that certain ART drugs may themselves have neurotoxic effects [12]. Furthermore, comorbidities frequently observed in PLWH may potentiate neurodegeneration through vascular and inflammatory pathways [12,13]. Despite these insights, few studies have comprehensively evaluated the impact of these variables on brain atrophy in a single cohort. This study aims to address this gap by systematically investigating the influence of demographic, behavioral, anthropometric, and comorbid factors on brain atrophy in PLWH and by developing a prediction model for brain atrophy in PLWH with multiple risk factors. A quantitative approach was employed to investigate brain atrophy by systematically evaluating 47 neuroimaging-derived metrics, representing cerebral substructures. By integrating multidimensional data, this work provides a better characterization of the determinants of brain aging in HIV, thereby informing strategies for early intervention, risk stratification, and personalized care in this vulnerable population.

## 2. Materials and Methods

### 2.1. Study Design

We performed a single-center cross-sectional study on 121 patients confirmed with HIV infection, recruited from the National Institute for Infectious Diseases, Prof. Dr. Matei Bals, Bucharest, Romania, between September 2023 and February 2025. The patients were further divided into two groups—Group A consisting of 38 patients with brain atrophy and Group B consisting of 83 patients without brain atrophy.

Inclusion criteria were as follows: positive diagnosis of HIV infection, documented duration of disease of minimum 10 years, age between 25 and 50 years, MRI cerebral exam performed during the hospital stay.

Exclusion criteria were as follows: MRI contraindications (claustrophobia, metallic implants/prosthesis, cardiac pace-maker, drug infusion pumps, cochlear implants, body piercings, metal fragments anywhere in the body), improper MRI image quality, cerebral surgery, stroke, cerebral masses (either benign or malign) depicted on MRI examination, chronic alcohol consumption (over 14 units weekly), history of intravenous/oral drug administration, age under 25 and over 50 years.

The study included also the evaluation of 96 patients, aged between 25 and 50 years, without any documented medical history as healthy controls. The exclusion criteria presented above were also applied for the control group. The study-group and the control-group were age-, sex-, and ethnicity-matched and they presented similar characteristics regarding the use of cigarettes and physical activity.

The study protocol respected the ethical and moral principles stated in the ‘Declaration on Human Rights’ in Helsinki, and it was approved by the local Ethics Committee of “Prof. Dr. Matei Balș National Institute for Infectious Diseases”. All patients were aged above 18 years, and gave their signed consent prior to the study enrollment.

For each patient in the study-group were quantified the demographic parameters (age, sex, ethnicity, and level of education), anthropometric parameters (weight, height, and BMI), smoker status, physical activity, duration of disease and of treatment. We registered the association of type 2 diabetes, chronic viral hepatitis, chronic renal disease, chronic lung disease, and HAND. For each patient were measured systolic and diastolic blood pressure values. All patients in our study received cART following a standardized national protocol, with only minor individual variations.

### 2.2. Imagistic Evaluation

The MRI evaluation was conducted using a Magnetom Sigma 3T system (SIEMENS, Erlangen, Germany), using a Head Neck 20TCS coil, with the following evaluation protocol: t1_fl2d_sag_4 mm, t2_tse_tra_512_4 mm, t2_tse_dark-fluid_tra_3 mm, resolve_4scan_trace_tra_p2_192_4 mm, t2_swi_tra_p2_2 mm, t1_fl2d_tra_4 mm, t2_tse_dark-fluid-cor_3 mm, and t1_mprage_tra_p2_iso_0.9 mm. The image post-processing was performed with the dedicated MR-Neurology software available on syngo-station (syngo—MRXA50A, SIEMENS, Erlangen, Germany). We quantified following parameters: total intracranial volume (TIV), brain volume (in mL and % from TIV), gray matter (in mL and % from TIV), cortical gray matter (in mL and % from TIV), white matter (in mL and % from TIV), cerebrospinal fluid (in mL and % from TIV), thalamus (in mL and % from TIV), putamen (in mL and % from TIV), caudate (in mL and % from TIV), globus pallidus (in mL and % from TIV), hippocampus (in mL and % from TIV), ventricles (in mL and % from TIV), frontal/parietal/occipital and temporal gray matter (in mL and % from TIV), insula, gyrus cinguli (in mL and % from TIV), frontal/parietal/occipital and temporal white matter (in mL and % from TIV), cerebellum (in mL and % from TIV) and corpus callosum (in cm^2^).

### 2.3. Definitions

Given the natural variability in total intracranial volume (TIV) among individuals, the diagnosis of brain atrophy was based on the percentage of specific brain region volumes relative to TIV rather than on absolute volume measurements. A threshold value of 79.1%—representing the lower limit of total brain volume as a percentage of TIV in the healthy control group—was established as the cut-off for identifying brain atrophy in patients with HIV infection.

The comorbidities were extracted from participants’ electronic medical records based on documented diagnoses made by attending specialists or primary care providers. The following operational definitions were used:

− type 2 diabetes: fasting plasma glucose ≥ 126 mg/dL on at least two occasions, HbA1c ≥6.5%, or current use of antidiabetic medication.
− systemic arterial hypertension: systolic blood pressure ≥ 140 mmHg and/or diastolic ≥ 90 mmHg on repeated clinic visits.
− chronic kidney disease: estimated glomerular filtration rate (eGFR) < 60 mL/min/1.73 m^2^ for at least 3 months.
− HAND: diagnosis made by a neurologist or infectious disease specialist using clinical neurocognitive testing, in accordance with the Frascati criteria.
  All comorbidities were recorded as binary variables (present/absent) and used in both univariate and multivariable analyses.

We calculated the body mass index (BMI) as weight (kg) divided by square height (m^2^). The patients were considered underweight for BMI lower than 18.5 kg/m^2^, normal for BMI between 18.5 and 24.9 kg/m^2^, overweight for BMI between 25 and 29.9 kg/m^2^, and obese for BMI over 30 kg/m^2^.

The physical activity was stratified in four categories based on the daily average estimated physical activity: 0 (under 15 min of continuous walk), 1 (15–30 min of continuous walk), 2 (30–60 min of continuous walk), 3 (over 60 min of continuous walk).

The history of smoking allowed stratification in non-smokers, ex-smokers, and active-smokers; for each patient the number of pack-years was quantified.

Based on the level of education we stratified the patients in three categories: (1) primary studies (less than 8 years), (2) high school (8–12 years), and (3) university/bachelor degree.

We considered compliance to treatment as existent in patients with values higher than 0.9 for the rapport between the duration of treatment and the duration of disease; this threshold is in concordance with both the WHO guidelines and the U.S. DHHS treatment recommendations cite 90–95% adherence as the benchmark for optimal clinical outcomes [14,15].

### 2.4. Statistic

For the data processing, we used the Statistical Package for Social Sciences (SPSS version 25, IBM Corp., Armonk, NY, USA). Patient data are presented as medians and quartiles (Q1, Q3) for the continuous variables and as percentages for the categorical variables. We used Mann–Whitney U test to evaluate the statistical differences for the MRI parameters between the HIV patients with and without brain atrophy.

We performed Spearman’s test to evaluate the correlation between the lifestyle, demographic, and comorbidity parameters and the presence of brain atrophy. Logistic regression analysis was performed to further characterize the relationship between brain atrophy and the registered parameters to obtain a brain atrophy prognostic model. The statistical significance of the final multivariable regression model was assessed using the Omnibus test of model coefficients. A *p*-value lower than 0.05 was considered statistically significant.

## 3. Results

### Study Group Characteristics

The patients in the study group included 68 males and 53 females, with a sex ratio male:female of 1.3:1 and a median age of 37 years [35; 47].

The distribution of the level of education among the patients was as follows: 32 (26.4%) completed primary education, 64 (52.9%) attained secondary education, and 25 (20.7%) achieved tertiary education.

Within the study cohort, smoking status was distributed as follows: 69 participants (57%) were classified as non-smokers, 34 (28.1%) as former smokers (mean smoking history: 8.3 pack–years), and 18 (14.9%) as active smokers (mean smoking history: 15.7 pack–years).

The patients presented a median height of 1.72 m [1.65; 1.78] and a median weight of 69 kg [60; 83]. We found 8 underweight patients (6.61%), 69 patients with normal weight (57.02%), 32 overweight patients (26.44%) and 12 obese patients (9.91%).

We registered 44 patients (36.36%) with less than 15 min of continuous walk daily, 53 patients (43.8%) between 15 and 30 min, 20 patients (16.52%) between 30 and 60 min and 4 patients (3.3%) with over 60 min of continuous walk daily.

The duration of disease was between 10 and 15 years for 61 patients (50.41%), between 15 and 20 years for 41 patients (33.88%) and over 20 years for 19 patients (15.7%), with 108 patients (89.25%) compliant to the antiretroviral treatment and 13 patients (10.74%) non-compliant to treatment.

We found diabetes type 2 in 17 cases (14.04%), arterial hypertension in 26 cases (21.48%), arterial hypotension in 3 cases (2.47%), chronic viral hepatitis in 15 cases (12.39%), chronic kidney disease in 12 cases (9.91%), chronic pulmonary disease in 13 cases (10.74%), and HAND in 39 cases (32.23%), registering higher percentages of comorbidities in Group A for all considered parameters. For the patients in Group A, HAND was the most frequent comorbidity (63.15%), while for the patients in Group B we found a higher percentage of patients with systemic arterial hypertension (19.27%) (Table 1).

In Group A, multimorbidity was more frequent, observing 14 patients (36.8%) with one comorbidity, 16 patients (42.1%) with two, 3 patients (7.9%) with three, and 1 patient (2.6%) with four comorbidities; 4 patients (10.5%) registered no comorbidities. In Group B, 37 patients (44.6%) had one comorbidity, 13 patients (15.7%) had two comorbidities, and 1 (1.2%) had three comorbidities; 32 patients (38.6%) presented no comorbidities.

The characteristics of the brain parameters measured in both Group A and B are further presented in Table 2.

Group A showed significantly smaller total brain volume (1166.7 vs. 1275.1 mL, *p* < 0.001) and lower brain percentage (72.9% vs. 83.1%, *p* < 0.001).

The patients with brain atrophy registered significant lower values for both gray matter (36.3% vs. 38.2% of TIV) and white matter (433.3 mL representing 27.5% vs. 506.1 mL representing 33.1% of TIV) and increased values for the cerebrospinal fluid (346.8 mL representing 22.2% vs. 257.6 mL representing 17.1% of TIV) in both percentage evaluation and absolute volume, with a more pronounced percentage reduction for the white matter compared to the total gray matter. The percentage of cortical gray matter relative to TIV was significantly lower in the brain atrophy group, despite no statistically significant difference in absolute cortical gray matter volume, highlighting the importance of using normalized measures (% of TIV) to detect parenchymal loss that may not be apparent when comparing absolute volumes alone, especially given the natural inter-individual variation in cranial size.

Analyzing the white matter atrophy, we can observe a reduction in the frontal (10.8% vs. 11.3% of TIV), parietal (6.8% vs. 7.2% of TIV) and temporal lobes (3.8% vs. 4.1%), and a smaller involvement of the occipital lobes (1.9% vs. 2.1% of TIV). The gray matter atrophy involved in our study the basal ganglia, with a significant reduction for the thalamus (0.97% vs. 1.03% of TIV) and putamen (0.96% vs. 1.02% of TIV) and the cortical gray matter in hippocampus (6.9% vs. 7.1% of TIV), frontal (13.7% vs. 14.4% of TIV) and parietal lobes (8.6% vs. 9.1% of TIV). Although, the cortical gray matter was also reduced in the temporal and occipital lobes, the differences presented no statistical significance.

We registered increased values for the cerebrospinal fluid with significant changes for the extranevraxial sector (322.8 mL vs. 236.5 mL); although the ventricles were larger for the patients in Group A (24mL), the differences were not significant compared to the patients in Group B (21.1mL).

We further evaluated the correlations between the presence of brain atrophy and the demographic, behavioral, anthropometric, and comorbid factors on brain atrophy in PLWH (Table 3).

We found significantly proportional correlations between the smoking status (0.395) and the number of pack–year (0.289) with the brain atrophy in PLWH. The physical activity (−0.323) inversely correlated with brain atrophy, with a higher risk for the patients with less than 30 min of continuous walking daily. The increased disease duration (0.416) and the lack of compliance with antiretroviral treatment (0.225) were also associated with the occurrence of brain atrophy. Regarding the documented comorbidities, we found significant correlations with brain atrophy in cases of type 2 diabetes (0.363) and HAND (0.448).

We performed ROC curves for the risk factors identified in Table 2 that presented significant correlations with the brain atrophy to better characterize their comparative performance (Table 4).

**Figure 1:**
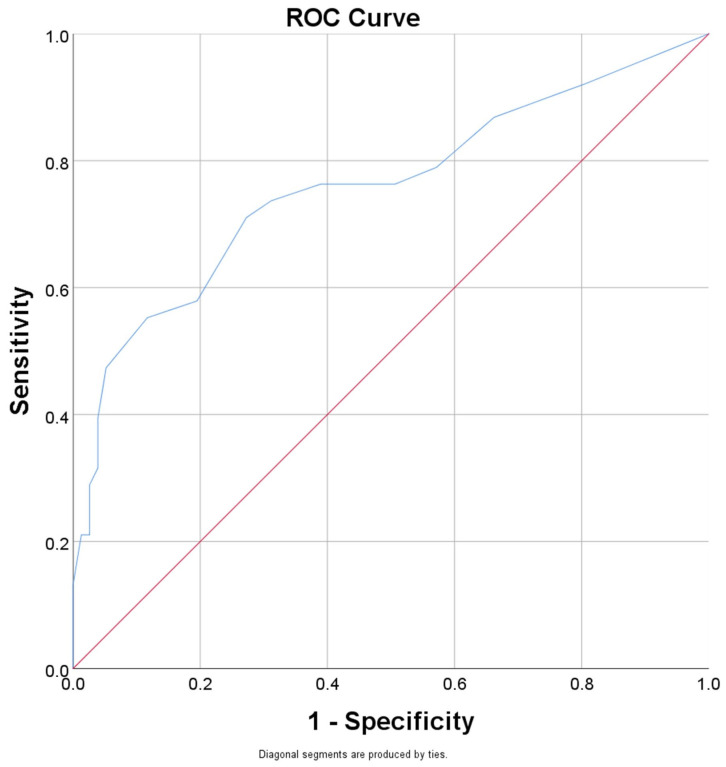
Receiver operating characteristics (ROC) curve for the ability of the “duration of disease” to predict brain atrophy. Diagonal reference line (red); ROC curve for the performance of “duration of the disease” (blue).

For the brain atrophy, we obtained the highest AUC for the parameter “disease duration” (Figure 1). Calculating Youden’s J for the duration of disease resulted in a value of 16.5 years, which can estimate the occurrence of brain atrophy with a sensitivity (Se) of 0.71 and a specificity (Sp) of 0.74.

A multivariable logistic regression analysis was subsequently conducted using the variables outlined in Table 3. The resulting model is shown in Table 5. According to the omnibus test of model coefficients, the model was statistically significant (*p* < 0.001) and demonstrated an overall prediction accuracy of 86.7%.

Based on the data in Table 5, we can also calculate the probability of brain atrophy in PLWH with multiple risk factors, using the following formula:

EXP(Constant + 1.662 × Smoking − 0.831 × Physical activity + 0.139 × Disease duration + 1.6 × Arterial hypertension + 2.418 × Chronic kidney disease) + 2.344 × HAND)/[1 + EXP (Constant + 1.662 × Smoking − 0.831 × Physical activity + 0.139 × Disease duration + 1.6 × Arterial hypertension + 2.418 × Chronic kidney disease) + 2.344 × HAND)].

## 4. Discussion

### 4.1. Prevalence and Patterns of Brain Atrophy

Brain (cerebral) atrophy represents a condition associated with decrease in brain parenchymal volume as a result of cells and neural connections loss [16,17]. Brain atrophy is detected, evaluated and quantified by using techniques as computed tomography and magnetic resonance imaging [12,18,19].

There are various factors that may induce neuronal and glial injury and promote cerebral atrophy: old age, infections, ischemia, cerebrovascular disease, head trauma, drugs and alcohol consumption, malnutrition, multiple sclerosis, cerebral palsy, and specific neurodegenerative diseases such as Alzheimer disease, Parkinson disease, etc. [17,20,21,22,23]. Evidence suggests that Galectin-3—a protein central to cell communication, immune regulation, and inflammation, and implicated in the pathology of neuroinflammatory and neurodegenerative diseases such as Alzheimer’s disease, Parkinson’s disease, and multiple sclerosis—also enhances HIV infection by cross-linking the virus to host cells and contributes to brain atrophy in PLWH [24,25,26,27,28].

Brain atrophy is typically assessed by detecting reductions in gray matter volume and increases in ventricular and sulcal dimensions [16]. Enlargement of the ventricles and sulci is indicative of central and cortical atrophy, respectively [16,29]. Several studies have reported significant increases in cerebrospinal fluid volume, as well as ventricular and sulcal enlargement, in association with brain atrophy [29,30,31]. In alignment with the existing literature, the present study demonstrated a significantly reduced total brain volume and a lower brain parenchymal fraction in Group A. Additionally, a significant increase in cerebrospinal fluid volume was observed within the extranevraxial compartment; although ventricular volumes were greater in Group A compared to Group B, this difference did not reach statistical significance.

Several distinct patterns of brain atrophy have been described, including focal, central, cortical, hemiatrophy, and diffuse atrophy [18]. Diffuse brain atrophy is characterized by widespread neuronal loss affecting all regions of the brain, commonly associated with aging, chronic substance use (alcohol or drugs), traumatic brain injury, infections, advanced stages of multiple sclerosis, and neurodegenerative disorders. In contrast, focal brain atrophy involves localized neuronal loss confined to specific brain regions, typically resulting from ischemic or hemorrhagic strokes, traumatic injury, or certain neurodegenerative diseases such as Alzheimer’s disease and Parkinson’s disease [18,32,33,34].

Brain atrophy is prevalent in approximately 61% of adults [35]. Focal and diffuse atrophy have been reported in 23.3% and 76.7% of individuals, respectively [33], while Imansyah et al. identified cortical atrophy in 54% and global atrophy in 11% of participants [19]. MRI studies in PLWH consistently demonstrate both diffuse and focal patterns of atrophy, with more extensive changes in individuals with HAND [31,36].

HIV-related brain injury is thought to follow a hierarchical pattern, initially affecting subcortical gray matter structures—such as the globus pallidus, putamen, and caudate nucleus—followed by white matter and subsequently the cortical gray matter [31,36]. The caudate nucleus is frequently implicated in neurocognitive decline [37], although some studies report more prominent putaminal atrophy [30,38], or even hypertrophy, potentially linked to inflammation, edema, or dopaminergic dysregulation [39,40]. Hippocampal atrophy is also a consistent finding and correlates strongly with cognitive impairment [30]. MRI remains essential for assessing neuroanatomical changes in PLWH and for elucidating HAND pathogenesis; Liu et al. reported significant atrophy of the hippocampus, thalamus, and occipital lobes [41].

In our study, patients with brain atrophy exhibited significantly lower gray and white matter volumes and increased cerebrospinal fluid volumes, with white matter reduction exceeding that of gray matter. White matter loss was most pronounced in the frontal, parietal, and temporal lobes, with relative sparing of the occipital lobes. Gray matter atrophy involved both subcortical (thalamus and putamen) and cortical structures (hippocampus, frontal, and parietal lobes), while reductions in the temporal and occipital cortices did not reach statistical significance.

Our finding that frontal, parietal, and temporal white-matter loss and thalamo-striatal gray-matter atrophy dominate the volumetric signature of brain aging in PLWH mirrors the pattern described in large North-American cohorts. For example, atrophy of the caudate and putamen persisted despite effective cART in the Multicenter AIDS Cohort Study (MACS) and was proportional to time since seroconversion [38], while the CHARTER study linked lower cortical GM and higher WM lesion burden to metabolic derangements rather than to current viral load [42]. Our data extend these observations to a younger, Eastern-European cohort (median age = 37 y) and confirm that subcortical vulnerability and fronto-subcortical WM loss remain evident even in populations with comparatively short infection duration and high treatment coverage.

### 4.2. Brain Atrophy and Behavioral Factors

Several studies have highlighted tobacco smoking as a major risk factor for pathological brain changes, with an even greater impact observed in PLWH. Reduced brain volumes, white matter abnormalities, and poorer executive function, even in individuals on suppressive ART have been associated with smoking, consistent with the results from our study [43,44,45]. Chronic smoking is associated with reduced cerebral perfusion, accelerate oxidative damage, and impair cerebral microstructure, mechanisms that are amplified in the context of HIV [46]. Mechanistically, smoking promotes endothelial dysfunction and disrupts the BBB, facilitating the entry of peripheral immune cells and viral proteins into the CNS; this process may enhance microglial activation, a key driver of neuroinflammation in neuroHIV [47,48]. Viral proteins reduce mitochondrial superoxide dismutase (Mn-SOD), weakening mitochondrial antioxidant defense. Tat activates NADPH oxidase (NOX4) in endoplasmic reticulum, raising hydrogen peroxide (H_2_O_2_) and promoting lipid peroxidation, whereas Tat and gp120 suppress synthesis of intracellular glutathione and its enzymes, augmenting intracellular reactive oxygen species (ROS) [49].

HIV infection has been shown to accelerate nicotine metabolism, potentially leading to increased tobacco use and more severe neuropathological consequences [50]. Smoking prevalence is higher among PLWH and appears to exert additive adverse effects on cognitive function, white matter integrity, and gray matter volume. The underlying mechanisms driving these changes are complex and not fully elucidated; however, preclinical studies suggest that both HIV infection and tobacco smoke exposure may promote heightened neuroinflammation, neurotoxicity, loss of myelin and brain atrophy, greater neuronal damage, and poor cognitive performance [12,48]. Moreover, cigarette smoking has been associated with higher viral loads and lower CD4+ T-cell counts, which may further contribute to the metabolic and body composition abnormalities increasingly observed in PLWH, particularly as this population experiences extended life expectancy and earlier onset of aging-related conditions [13,48,51].

PLWH develops faster musculoskeletal aging, particularly sarcopenia and osteoporosis with decreased physical activity [52]. In the present study, physically active HIV-positive individuals exhibited significantly less brain atrophy compared to their sedentary counterparts, aligning with previous studies investigating the relationship between physical activity and brain structural integrity in HIV-infected populations. Physical activity may exert direct neuroprotective effects by upregulating growth factors expression and enhancing neuronal survival. Indirectly, it may improve metabolic regulation and attenuate inflammatory processes by enhancing cardiorespiratory fitness and skeletal muscle function [53,54]. Reduced physical activity may exacerbate brain atrophy through both direct neurotrophic deficits and indirect metabolic and inflammatory mechanisms. Physical exercise upregulates brain-derived neurotrophic factor (BDNF), promotes hippocampal neurogenesis, and improves synaptic plasticity, all of which contributes to the maintenance of brain volume and cognitive function [55]. In HIV-infected individuals, these protective effects may be particularly relevant due to baseline elevations in neuroinflammation and oxidative stress. Evidence suggests that exercise reduces peripheral inflammatory cytokines (e.g., IL-6, IL-5, IL-8, IL-10, TNF-α) in PLWH, which are known to cross the blood–brain barrier (BBB) and contribute to glial activation and neurotoxicity [56,57]. Furthermore, regular exercise enhances insulin sensitivity, potentially reducing the risk of diabetes-related microvascular brain injury, which we also found to be a major risk factor in our cohort.

Several accelerometry and cohort studies demonstrate that even light physical activity correlates with larger total GM and faster processing speed in middle-aged/older PLWH and preserves global cognition longitudinally [58,59].

By integrating these variables, our multivariable model highlights concrete behavioral targets (smoking cessation, ≥30 min/day continuous walking) that could mitigate atrophy risk.

Antiretroviral therapy, which has significantly improved life expectancy for PLWH, is frequently associated with the development of metabolic syndrome. This syndrome is linked to an increased risk of type 2 diabetes and cardiovascular disorders. Among the key features of metabolic syndrome, obesity and type 2 diabetes are well-established risk factors for neurocognitive impairment [60]. Specific studies have identified central obesity (rather than generalized increased BMI) and type 2 diabetes—particularly in older patients—as being associated with a higher prevalence of neurocognitive impairment in HIV-positive individuals [61].

We report a data-driven cut-off of ≥16.5 years of infection that yields 71% sensitivity and 74% specificity for predicting brain atrophy. Although disease duration is routinely recorded, the present threshold provides an actionable benchmark that is rarely formalized in previous work. The MACS basal-ganglia analysis showed a near-linear relationship between years since seropositivity and striatal shrinkage without proposing a practical cut-point [38]. Longer follow-up studies have confirmed continuing volumetric loss after more than 15 years of cART, accompanied by neuro-inflammatory CSF signatures [62]. Our ROC-derived threshold therefore refines these longitudinal observations into a clinically usable screening indicator. Although our patients were comparatively young, the trajectory we report is consistent with evidence that age amplifies HIV-associated WM hyperintensities and FA decline, preferentially in fronto-subcortical tracts [63]. This synergy reinforces the urgency of early lifestyle and vascular interventions well before mid-life. The present study demonstrates a significant correlation between brain atrophy and disease duration, corroborating previous research that identified overall cerebral volume—including gray and white matter, as well as volumes of the parietal, temporal, and frontal lobes, and the hippocampus—as being most strongly associated with the duration of HIV infection and nadir CD4+ lymphocyte count [64]. These findings suggest that a history of severe immune dysfunction, as evidenced by a low nadir CD4+ count in HIV-infected individuals, is strongly linked to brain volume loss and an increased risk of cerebral atrophy.

Early-treated patients showed caudate-putamen loss over the two years following acute infection [65] whereas a Canadian longitudinal case-control study found no additional atrophy over two years in well-suppressed individuals but confirmed baseline deficits in cortical thickness and subcortical volume [4]. Collectively, these data and our cross-sectional results indicate that a sizeable fraction of brain damage occurs before—and only partly normalizes after—sustained viral suppression, justifying intensified surveillance even in apparently stable patients.

Studies have shown that central nervous system inflammation can persist even in the presence of effective cART, suggesting that cART alone may not be sufficient to prevent ongoing brain damage [66]. Despite achieving viral suppression, the persistence of HIV DNA in brain tissue of virally suppressed individuals on cART has been demonstrated, which may account for findings indicating that long-term suppressed HIV-infected individuals experience brain atrophy rates that exceed those observed in healthy controls [67]. In our study, we identified a significant association between non-adherence to the antiretroviral treatment and the occurrence of brain atrophy, underscoring the importance of achieving viral suppression and adequate immunological recovery with effective cART to slow the progression of brain atrophy [68].

In addition to examining the relationships between brain tissue loss and indicators of disease severity—such as immunological markers, including baseline CD4+ cell count and the duration of HIV infection—demographic factors were also assessed for their associations with changes in regional brain volumes and neurocognitive impairment. In our study, no significant correlation was found between brain atrophy and education level, consistent with findings from other studies. This may be explained by the fact that most study participants had at least a high school education [69].

### 4.3. Comorbidities in PLWH and Brain Atrophy

The processes contributing to brain aging in PLWH are multifactorial, involving the direct neuropathological effects of the virus, sustained systemic inflammation, antiretroviral therapy (ART)-associated neurotoxicity, cerebrovascular alterations, and the presence of comorbid conditions, all of which interact with and exacerbate normative age-related neurobiological changes [70].

Approximately 42.6% of PLWH experience neurocognitive impairment, which adversely impacts quality of life, adherence to treatment, employment status, and overall survival [71]. In the present study, HAND was identified in 39 individuals, accounting for 32.23% of the total cohort. Notably, a significant association was observed between the presence of HAND and the occurrence of brain atrophy. According to the Frascati criteria, HAND is categorized into three distinct clinical entities based on severity: asymptomatic neurocognitive impairment (ANI), mild neurocognitive disorder (MND), and HIV-associated dementia (HAD) [12,72]. The pathogenesis of HAND is associated with synaptodendritic degeneration and neuronal damage, driven by viral proteins such as gp120, gp41, Tat, Vpr, Nef, and Rev, along with proinflammatory cytokines and chemokines released by activated microglia. Additionally, elevated extracellular glutamate also contributes to bioenergetic dysfunction [12,72]. HIV impairs the integrity and functionality of the blood–brain barrier (BBB) by diminishing pericyte coverage and disrupting intercellular signaling pathways, thereby affecting interactions with endothelial cells [12,73]. The compromise of the BBB reduces the transport of essential nutrients to the central nervous system while facilitating the entry of pathogens, immune cells, and neurotoxic substances, which collectively contribute to accelerated brain aging in PLWH [12,74]. HIV infection of microglial cells enhances the release of neurotoxic mediators, thereby accelerating the normal decline in myelination and contributing to white matter volume loss [12,75]. Additionally, astrocyte infection plays a significant role in sustaining neuroinflammation and is implicated in the development of HAND [12,76]. Furthermore, HIV is linked to disruptions in neurogenesis and telomere shortening, both of which exacerbate the progression of premature brain aging [12].

Current studies demonstrate a high incident of type 2 diabetes in PLWH ranging from 4.3% [77] to 26.8% [78], similar to the values found in our study (14.04%), with higher percentage in PLWH that associate brain atrophy (21.05%). Reduced glucose availability alongside heightened glucose utilization is key features of the aging brain. In PLWH, there is a rise in oxidative stress and a decline in processes such as oxidative phosphorylation, gluconeogenesis, ATP generation, and beta-oxidation. These individuals also experience disrupted cellular homeostasis, increased mitochondrial DNA mutations, and elevated levels of apoptosis. Ongoing mitochondrial damage, caused by both HIV infection and antiretroviral therapy, disrupts mitochondrial function, leading to greater energy deficits, faster cellular aging, senescence, and impaired cell function [12]. Patients with chronic hyperglycemia may associate a higher degree of atrophy, which requires better control and a stricter diet of diabetic patients. Our finding that type 2 diabetes conferred a 5.8-fold increase in atrophy odds converges with a meta-analysis of >31,000 individuals reporting 1–4% reductions in total, GM, WM, and hippocampal volumes in diabetes, independent of HIV [79]. Within the CHARTER cohort, hyperglycaemia and other metabolic-syndrome traits were likewise linked to greater abnormal WM volume [42], underlining that cardiometabolic optimization remains integral to neuroprotection in treated HIV.

Diabetes control can be more difficult in PLWH taking into consideration possible reduced compliance of patient and the pharmacological interactions between the ARV molecules and the oral antidiabetic medication (higher risk of lactic acidosis induced by metformin, more severe lipodystrophy, more frequent hypoglycemia, irritative digestive symptoms) [80]. In PLWH with metabolic syndrome, significant increases in cerebrospinal fluid volume, as well as enlargement of the lateral ventricles, have been observed when compared to PLWH without metabolic syndrome; these changes occurred independently of age-related brain volume reductions and despite effective HIV suppression [81]. Consistent with these findings, the present study revealed significant correlations between brain atrophy and type 2 diabetes in PLWH. The mechanisms underlying these associations remain unclear; however, the ongoing secretion of inflammatory mediators by adipocytes, insulin resistance, and the release of adipose tissue-derived hormones are potential contributors to the neurodegenerative processes that lead to cognitive dysfunction [61].

Systemic arterial hypertension represents a frequent comorbidity found in up to 56% of PLWH [82], also observed in our study group in 21.48% of cases. Recent studies demonstrated that HIV Tat protein accelerates the production of beta-amyloid and contributes to a progressive decrease in vascular lumen, with a reduction in cerebral blood flow and chronic cerebral hypoperfusion [83,84]. Although, the systemic arterial hypertension was registered more frequently in the PLWH with brain atrophy compared to PLWH without brain atrophy (26.31% vs. 19.27%), we found no significant correlation between brain atrophy and arterial hypertension in our study. A possible explanation is that the PLWH develop brain atrophy mostly secondary to the disruption of the structure and function of the blood–brain barrier (BBB) and not directly to increased blood pressure. The involvement of BBB implies reduced pericyte coverage of the vessels, altering cellular signaling, and their interaction with the endothelial cells [85]. The loss of BBB integrity induces the reduction in vital nutrients entering the brain and the reduction in the blockage of pathogens, inflammatory cells, and other toxic agents, leading to early brain aging, and cerebral atrophy [75].

The percentage of PLWH with chronic hepatitis (B or C) coinfection ranges from approximately 10% to 50% worldwide, depending on the geographic region, representing one of the most frequent comorbidities [85,86]. Patients with chronic hepatitis may associate metabolic syndrome, augmented by the antiviral treatment, characterized by mitochondrial dysfunction, insulin resistance, and accumulation of proinflammatory and pro-coagulant mediators with systemic consequences, augmenting the inflammatory status induced by HIV infection and amplify its consequence [87,88]. In patients with advanced chronic hepatitis, accumulation of toxic substances like manganese or ammonia secondary to defective liver clearance mechanisms may facilitate development of acquired hepatocerebral degeneration [89]. In our study we found chronic hepatitis in 15 cases, representing 12.39% of patients, all of them presenting compensated forms of chronic hepatitis, which may explain the lack of association with the brain atrophy due to a low systemic impact. Prevalence of chronic kidney disease (CKD) in PLWH has a wide range of variation from 2.3% to 53.3%, mostly explained by the multiple risk factors for the sample populations [90]. Correlations between cerebral atrophy and CKD have been documented especially in dialysis patients, associated with vascular involvement and accumulation of toxic metabolites [91]. In our study, we found CKD in 12 cases (9.91%), none of them necessitating dialysis, which can explain the lack of significant association with brain atrophy.

### 4.4. Study Limitations and Future Research Directions

The threshold for brain atrophy in our study was internally generated to ensure methodological consistency and reduce potential variability introduced by external normative datasets; however, further validation in larger and independent populations—ideally with normative brain volume datasets—is needed to generalize this threshold beyond the current cohort. Additional intercurrent pathologies or socio-economic factors (not considered in this study) may also influence the evolution of brain atrophy. Comorbidities were recorded as binary variables, due to limitations in retrospective data availability and to reduce variability in definitions across patients. Opportunistic infections and prior AIDS-defining illnesses were not available for all patients and were therefore not included in the analysis. The small sample of the cohort study requires further validation in larger and ideally longitudinal datasets with external validation for both risk factors and the prediction model for brain atrophy in PLWH. The developed formula for brain atrophy should be interpreted as exploratory and hypothesis-generating, requiring further validation in longitudinal studies. The cross-sectional nature of our study allows us to establish demonstrated associations, not causation, between risk factors and brain atrophy in PLWH. Exploring broader age ranges in future studies, including longitudinal designs that can capture age-related trajectories of neurodegeneration.

## 5. Conclusions

The present study offers a comprehensive analysis of brain atrophy in PLWH in Romania, highlighting significant associations with smoking, reduced physical activity, and the duration of disease. We can estimate the occurrence of brain atrophy with a sensitivity (Se) of 0.71 and a specificity (Sp) of 0.74 for PLWH with a duration of the disease of 16.5 years. Type 2 diabetes and HAND are also strongly associated with brain atrophy. These findings underscore the importance of comprehensive management strategies for PLWH, encompassing not only viral suppression but also lifestyle modifications and management of comorbid conditions. Regular monitoring of cognitive function and brain imaging may be warranted in individuals with identified risk factors to facilitate early intervention.

## Figures and Tables

**Table 1:** Comorbidities in the study groups.

Parameter	Group A (38)		Group B (83)	
	Number of Cases	Percentage	Number of Cases	Percentage
Type 2 diabetes	8	21.05%	9	10.84%
Arterial hypertension	10	26.31%	16	19.27%
Arterial hypotension	1	2.63%	2	2.41%
Chronic hepatitis	4	10.52%	11	13.25%
Chronic kidney disease	6	15.78%	6	7.23%
COPD	6	15.78%	7	8.43%
HAND	24	63.15%	15	18.07%

Abbreviations: COPD, chronic obstructive pulmonary disease, HAND, HIV-associated neurocognitive disorder.

**Table 2:** Brain parameters in Group A and Group B.

Parameter	Group A	Group B	*p*-Value
Brain (mL)	1166.7 [1102.5;1201.2]	1275.1 [1211.2; 1341.8]	<0.001
Brain (% *)	72.9 [72.1; 74.4]	83.1 [81.2; 84.6]	<0.001
Gray matter (mL)	720.3 [650.1; 762.2]	752.1 [706; 812.8]	0.003
Gray matter (% *)	45.1 [41.3; 47.2]	49.4 [45.6; 52.7]	<0.001
Cortical gray matter (mL)	563.8 [520; 626.3]	582.8 [529.3; 633.9]	0.326
Cortical gray matter (% *)	36.3 [33.1; 38.7]	38.2 [35.1; 41.3]	0.006
White matter (mL)	433.3 [393.9; 494.6]	506.1 [471.1; 568.9]	<0.001
White matter (% *)	27.5 [26.1; 32.1]	33.1 [31.3; 35.8]	<0.001
Cerebro-spinal fluid (mL)	346.8 [315.1; 372.9]	257.6 [235.1; 307.3]	<0.001
Cerebro-spinal fluid (% *)	22.2 [21.3; 22.6]	17.1 [15.4; 19.2]	<0.001
Thalamus (mL)	14.9 [13.9; 16.4]	15.5 [14.4; 16.5]	0.096
Thalamus (% *)	0.97 [0.89; 1]	1.03 [0.96; 1.08]	<0.001
Putamen (mL)	15 [14.2; 15.8]	15.7 [14.4; 16.6]	0.085
Putamen (% *)	0.96 [0.91; 0.98]	1.02 [0.96; 1.07]	<0.001
Caudate (mL)	9.1 [8.6; 10.1]	9.23 [8.37; 10.14]	0.806
Caudate (% *)	0.6 [0.54; 0.64]	0.62 [0.56; 0.67]	0.290
Globus pallidus (mL)	4.3 [4; 4.7]	4.1 [3.8; 4.5]	0.105
Globus pallidus (% *)	0.28 [0.25; 0.3]	0.27 [0.26; 0.3]	0.698
Hippocampus (mL)	6.9 [6.4; 7.5]	7.1 [6.4; 7.7]	0.250
Hippocampus (% *)	0.43 [0.41; 0.47]	0.47 [0.43; 0.5]	0.008
Ventricles (mL)	24 [19.1; 27.3]	21.1 [18.3; 25]	0.144
Ventricles (% *)	1.5 [1.2; 1.8]	1.4 [1.2; 1.6]	0.490
Frontal gray matter (mL)	214.9 [198.1; 233.3]	220 [202; 240.2]	0.208
Frontal gray matter (% *)	13.7 [12.7; 14.3]	14.4 [13.3; 15.6]	0.002
Parietal gray matter (mL)	131.7 [115; 152.7]	137.1 [123.4; 153.4]	0.369
Parietal gray matter (% *)	8.6 [7.5; 9.2]	9.1 [8.2; 9.9]	0.031
Occipital gray matter (mL)	67.95 [62.9; 69.9]	67.9 [60.6; 75.1]	0.619
Occipital gray matter (% *)	4.3 [4; 4.5]	4.5 [4; 4.7]	0.051
Temporal gray matter (mL)	142.6 [126.6; 168]	142.9 [128.1; 155]	0.617
Temporal gray matter (% *)	9.1 [7.9; 10.4]	9.3 [8.3; 10.2]	0.647
Insular gray matter (mL)	13.5 [12.6; 14.3]	12.9 [12.2; 13.8]	0.087
Insular gray matter (% *)	0.87 [0.8; 0.92]	0.85 [0.81; 0.92]	0.748
Cingular gray matter (mL)	19.8 [17.4; 20.7]	19.2 [17.3; 20.5]	0.657
Cingular gray matter (% *)	1.2 [1.1; 1.3]	1.2 [1.2; 1.3]	0.071
Frontal white matter (mL)	172.5 [153.4; 185.5]	173.6 [157.3; 189.3]	0.366
Frontal white matter (% *)	10.8 [10.1; 11.5]	11.3 [10.6; 12.1]	0.003
Parietal white matter (mL)	102.5 [93.5; 113.9]	107.6 [98.9; 123.4]	0.056
Parietal white matter (% *)	6.8 [6.1; 7.1]	7.2 [6.6; 7.8]	0.002
Occipital white matter (mL)	29.5 [25.2; 36.3]	32.3 [26; 36.2]	0.490
Occipital white matter (% *)	1.9 [1.6; 2.3]	2.1 [1.7; 2.4]	0.064
Temporal white matter (mL)	59.4 [54.7; 64.2]	62.7 [57.3; 65.1]	0.058
Temporal white matter (% *)	3.8 [3.6; 4]	4.1 [4; 4.1]	<0.001
Cerebellum (mL)	134.6 [128.4; 144.1]	134.8 [127.9; 143.8]	0.891
Cerebellum (% *)	8.5 [8.2; 9.2]	8.8 [8.5; 9.4]	0.075
Corpus callosum (cm^2^)	5.2 [4.9; 5.5]	5.2 [5; 5.6]	0.893

Note: * all the percentage parameters are reported to the total intracranial volume (TIV).

**Table 3:** Correlations between brain atrophy and demographic, behavioral, anthropometric, and comorbid risk factors.

Parameter	Spearman’s Rho	*p*-Value	OR	[CI]
Education	−0.101	0.271	0.730	[0.413; 1.290]
Smoking status	0.395	<0.001	3.395	[1.921; 6]
Number of pack-year	0.289	<0.001	1.074	[1.018; 1.132]
Weight	0.103	0.261	1.015	[0.992; 1.039]
Height	0.061	0.505	6.710	[0.135; 332.439]
BMI	0.100	0.273	1.043	[0.951; 1.144]
Physical activity	−0.323	<0.001	0.366	[0.200; 0.669]
Disease duration	0.416	<0.001	1.244	[1.127; 1.372]
Treatment duration	0.394	<0.001	1.275	[1.137; 1.430]
Lack of compliance to treatment	0.225	0.013	4.160	[1.260; 13.730]
Type 2 diabetes	0.363	<0.001	5.829	[2.323; 14.626]
Arterial hypertension	0.080	0.386	1.496	[0.605; 3.696]
Arterial hypotension	0.007	0.943	1.095	[0.096; 12.455]
Chronic hepatitis	−0.038	0.676	0.770	[0.229; 2.595]
Chronic kidney disease	0.110	0.229	2.406	[0.722; 8.024]
COPD	0.110	0.227	2.036	[0.634; 6.533]
HAND	0.448	<0.001	7.771	[3.274; 18.445]

Abbreviations: CI, confidence interval, COPD, chronic obstructive pulmonary disease, HAND, HIV-associated neurocognitive disorder; OR, odds ratio.

**Table 4:** ROC curve analysis for the parameters associated with brain atrophy.

Parameter	AUC	Std Error	*p*-Value	CI 95%	
				Lower Bound	Upper Bound
Smoking status	0.718	0.053	<0.001	0.615	0.822
Number of pack-year	0.661	0.054	0.005	0.554	0.768
Physical activity	0.313	0.053	0.001	0.210	0.416
Disease duration	0.757	0.052	<0.001	0.655	0.860
Lack of compliance to treatment/Non-adherence	0.575	0.058	0.186	0.461	0.690
Diabetes type II	0.663	0.057	0.004	0.551	0.774
HAND	0.725	0.053	<0.001	0.622	0.829

Abbreviations: AUC, area under curve; CI, confidence interval; HAND, HIV-associated neurocognitive disorders.

**Table 5:** Multivariable logistic regression model for patients with brain atrophy.

Variable	B	S.E.	Wald	*p*	OR	95% CI for OR	
						Lower	Upper
Smoking	1.662	0.473	12.340	<0.001	5.271	2.085	13.327
Physical activity	−0.831	0.408	4.145	0.042	0.436	0.196	0.969
Disease duration	0.139	0.057	6.017	0.014	1.149	1.028	1.284
Arterial hypertension	1.600	0.799	4.007	0.045	4.955	1.034	23.741
Chronic kidney disease	2.418	0.852	8.058	0.005	11.220	2.114	59.565
HAND	2.344	0.664	12.456	<0.001	10.425	2.836	38.322
Constant	−4.974	1.134	19.230	<0.001	0.007		

Abbreviations: B, regression coefficient; CI, confidence interval; HAND, HIV associated neurocognitive disorder; OR, odds ratio; S.E, standard error of the coefficient.

## Data Availability

The data supporting the conclusions of this article will be made available from the authors on request.
